# Type III restriction-modification enzymes: a historical perspective

**DOI:** 10.1093/nar/gkt616

**Published:** 2013-07-17

**Authors:** Desirazu N. Rao, David T. F. Dryden, Shivakumara Bheemanaik

**Affiliations:** ^1^Department of Biochemistry, Indian Institute of Science, Bangalore 560 012, India and ^2^School of Chemistry, The King’s Buildings, The University of Edinburgh, Edinburgh EH9 3JJ, Scotland, UK

## Abstract

Restriction endonucleases interact with DNA at specific sites leading to cleavage of DNA. Bacterial DNA is protected from restriction endonuclease cleavage by modifying the DNA using a DNA methyltransferase. Based on their molecular structure, sequence recognition, cleavage position and cofactor requirements, restriction–modification (R–M) systems are classified into four groups. Type III R–M enzymes need to interact with two separate unmethylated DNA sequences in inversely repeated head-to-head orientations for efficient cleavage to occur at a defined location (25–27 bp downstream of one of the recognition sites). Like the Type I R–M enzymes, Type III R–M enzymes possess a sequence-specific ATPase activity for DNA cleavage. ATP hydrolysis is required for the long-distance communication between the sites before cleavage. Different models, based on 1D diffusion and/or 3D-DNA looping, exist to explain how the long-distance interaction between the two recognition sites takes place. Type III R–M systems are found in most sequenced bacteria. Genome sequencing of many pathogenic bacteria also shows the presence of a number of phase-variable Type III R–M systems, which play a role in virulence. A growing number of these enzymes are being subjected to biochemical and genetic studies, which, when combined with ongoing structural analyses, promise to provide details for mechanisms of DNA recognition and catalysis.

## INTRODUCTION

The molecular basis of restriction and modification (R–M) of DNA was first described in 1962, when Arber and Dussoix explored the host-controlled modification of bacteriophage λ, a genetic phenomenon that had been known for about a decade (1). ‘Host specificity’ (restriction–modification or R–M) was explained in molecular terms as an endonucleoytic cleavage of foreign DNA. Cellular DNA was protected from restriction by modification (methylation) of adenosyl or cytosyl bases within defined recognition sequences. Thus, R–M systems are composed of pairs of opposing enzyme activities: a restriction endonuclease (REase) and a DNA methyltransferase (MTase). Based on their molecular structure, sequence recognition, cleavage position and cofactor requirements, R–M systems are currently classified into three groups–Types I, II and III with a fourth group, Type IV, showing restriction of modified DNA (2).

Type III R–M systems are present in most sequenced bacterial genomes, and >1600 putative Type III R–M systems are known (http://rebase.neb.com/cgi-bin/azlist?re3). Among these, the recognition sequences for ∼60 Type III R–M enzymes have been determined. Though only a handful of these enzymes have been biochemically characterized, the presence of these R–M systems in thousands of bacteria indicates their importance to these organisms. Therefore, understanding these enzymes should give an insight into the roles that these R–M systems play in host biology and the reasons for their evolution and maintenance.

This review focuses on the small group of well-characterized Type III R–M enzymes (Table 1), and most knowledge comes from studies on the enzymes from the bacteriophage P1 and the related p15B plasmid, which have *Escherichia coli* as their host. The EcoP1 and EcoP15 R–M systems (formally called EcoP1I and EcoP15I) are the only ones that have been extensively studied. The review will largely follow a chronological pattern from early genetics through biochemical analysis to modern structural and single-molecule experiments and biotechnological uses.

**Table 1.** Properties Type III restriction enzymes

Enzyme	Source	Recognition sequence	Subunit composition	Cofactor requirement for restriction	Cofactor requirement for methylation
EcoP15I	Plasmid P15B in *E. coli* 15T^−^	5′-CAGC**A**G-3′	R_2_ M_2_	Mg^2+^, ATP, (SAM?)	SAM, Mg^2+^
EcoP1I	Prophage P1	5′-AG**A**CC-3′	R_2_ M_2_	Mg^2+^, ATP, (SAM?)	SAM, Mg^2+^, Ca^2+^, Mn^2+^
StyLTI	*Salmonella typhimurium*	5′-CAG**A**G-3′	R_2_ M_2_	Mg^2+^, ATP, SAM	SAM, Mg^2+^
HinfIII	*Haemophilus influenzae*	5′-CG**A**AT-3′	R_2_ M_2_	Mg^2+^, ATP	SAM, Mg^2+^
PstII	*Providencia stuartii*	5′-C**A**TCAG-3′	R_2_ M_2_	Mg^2+^, ATP, GTP, CTP	SAM, Mg^2+^
LlaFI	*Lactococcus lactis*	ND	ND	Mg^2+^, ATP	ND
BceSI	*Bacillus cereus*	ND	ND	Mg^2+^, ATP	SAM
PhaBI	*Pasteurella haemolytica*	ND	ND	ND	ND
NgoAXP	*Neisseria gonorrhoeae*	5′-CC**A**CC-3′	ND	ND	SAM, Mg^2+^
HP0593	*Helicobacter pylori*	5′-GC**A**G-3′	ND	ND	SAM, Me^2+^

ND, not determined.

**A** denotes site of methylation.

## EARLY EXPERIMENTS ON TYPE III R–M SYSTEMS

### Efficiency of plating of phage λ on phage P1 lysogens of *Escherichia coli*

**Figure 1.**
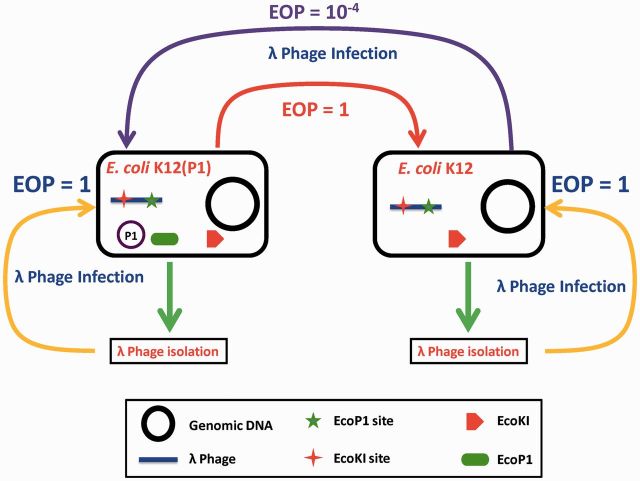
Schematic illustration showing the presence of an R–M system in P1 prophage. This cartoon depicts the infection of λ phage isolated from *E. coli* K12 strain into *E. coli* K12(P1) lysogens and vice versa. The infection of an *E. coli* K12(P1) lysogen with λ phage isolated from *E. coli* K12 resulted in reduced e.o.p. However, infection of *E. coli* K12 with λ phage isolated from the *E. coli* K12(P1) lysogen was efficient. These experiments suggested that P1 phage harbors an independent R–M system, later identified as EcoP1.

Genetic determinants for R–M activities are carried by many bacterial strains, but they were most extensively studied during the 1960s using four *E. coli* strains: K12, B, 15T^−^ and the K12(P1) lysogen. Strains K12, B and 15T^−^ carry the chromosomal EcoK, EcoB and EcoA Type I R–M systems, respectively. Arber and Dussoix (1) in Geneva described host specificity conferred on λ phage after infection of an *E. coli* K12(P1) lysogen. Phage λ recovered from K12 was restricted when subsequently plated on the K12(P1) lysogen. However, any recovered phage were fully biologically active when replated on the K12(P1) lysogen (Figure 1), indicating that they had acquired a modification rendering them resistant to P1 restriction. This agreed with previous data showing that restriction of phage λ recovered from K12 was more severe when plated on the P1 lysogen than on the non-lysogenic *E. coli* K12 and suggested that there were two independent R–M systems in the lysogen and only one in the non-lysogenic strain (1). The modification acquired by the phage recovered from the lysogen was lost after subsequent passage through a non-lysogenic strain. Other experiments using conjugation (3) and transformation or transduction (4) soon supported the notion that phage P1 harbored its own R–M system distinct from the EcoK, EcoB and EcoA chromosomal R–M systems.

### Experiments with *Escherichia coli* 15T^−^ and the discovery of the EcoP15 R–M system on plasmid P15B

Conjugation studies between a male derivative of *E. coli* 15T^−^ and *E. coli* K12 females by Kenneth Stacey showed that *E. coli* 15T^−^ strains had an R–M system distinct from the EcoK R–M system in strain K12 (5). This observation was supported by Arber and Linn (6) who found a second set of R–M genes were present on a plasmid. This R–M system was closely related to the R–M system of phage P1. The close relationship was shown by its recombination with P1 and by its competition with P1 for stable inheritance. A year later, Arber and Wauters-Williems (7) clearly showed that *E. coli* 15T^−^ had two distinct sets of R–M systems. The genetic information for system A (EcoA) was carried on the bacterial chromosome as aforementioned. This Type I R–M system was linked to the *thr* region on the chromosome and genetically related to the EcoK and EcoB R–M systems in the K12 and B strains.

Among the phages that were tested, phage 82 grew well on the *E. coli* 15T^−^ strain. However, if previously passaged on host strains K12 or B, it then grew poorly on *E. coli* 15T^−^ with an efficiency of plating (e.o.p.) of ∼10^−^^4^ to 10^−^^5^. When *E. coli* K12 and *E. coli* B strains were infected with phage 82 isolated from the *E. coli* 15T^−^ strain, the phages were similarly restricted. However, when *E. coli* 15T^−^ strain was infected with phage 82 isolated from *E. coli* 15T^−^ strain, no restriction was observed. As the EcoA R–M system was on the chromosome of *E. coli* 15T^−^, it was concluded that the plasmid p15B harbored another R–M system, which was named the EcoP15 R–M system (7).

Arber’s work also showed that the p15B plasmid of *E. coli* 15T^−^ could genetically recombine with the genome of phage P1 and that they had the same function in controlling stable inheritance. Much of the work on the EcoP15 R–M system was done in parallel with the EcoP1 R–M system, as both systems share a great degree of homology, and each could complement mutations in the other system (7). It was observed that the stable inheritance of the p15B plasmid was disturbed on P1 lysogenisation. Loss of the plasmid from these strains was a consequence of P1 superinfection. Furthermore, the streptomycin-resistant mutations that affected EcoP1 restriction had the same effect on EcoP15 (8,9).

In addition, these studies showed that the restriction effects of EcoA and EcoP15 simultaneously carried by *E. coli* 15T^−^ were additive in the same sense, as EcoK and EcoP1 restriction were additive in P1-lysogenic strains of K12. However, mutants of EcoA or EcoK could not be complemented by the P1 or P15 systems; thus, there was no interaction between the EcoA/EcoK enzymes with the EcoP1/EcoP15 enzymes (7). The P1 and P15 R–M systems were eventually designated by Andrej Piekarowicz in 1978 as the Type III R–M systems (10) to distinguish them from the Type I and the Type II systems.

### A role for methionine in the generation of host specificity

The nature of the modification acquired by phage recovered from the P1 lysogen was determined by the use of methionine mutants (met^−^) of *E. coli* K12, B and K12(P1) (4). The mutation led to a deficiency in the amount of S-adenosyl-L-methionine (SAM) synthesized and inhibition of the modification function of the R–M system. All three strains were grown to log phase, infected with phage λ, starved for methionine for a fixed period and then growth resumed by the reintroduction of the missing amino acid. Cells were lysed with chloroform, the λ lysate plated on *E. coli* C (a strain lacking R–M completely, a phenotype of r^−^m^−^) and the host strain (r^+^m^+^) to determine the extent of modification. Deprivation of methionine during the startvation period resulted in the production of phage showing a lower e.o.p. on the host strain than on the *E. coli* C strain. This lower e.o.p. was comparable in extent on the B, K and K12(P1) strains and was indicative of lower levels of modification of the phage DNA by the EcoB, EcoK and EcoP1 R–M systems. It was concluded that modification required SAM.

A further indication of the importance of SAM came from experiments on the growth of *E. coli* in the presence of methionine analogs such as ethionine and norleucine. These experiments showed the breakdown of cellular DNA in *E. coli* 15T^−^ and P1 lysogens of other strains but not in strains lacking the P15 or P1 systems (11). Lark and Arber (11) showed that in the strains with EcoP1 and EcoP15, blocking synthesis of SAM with the methionine analogs did not inhibit DNA synthesis, but newly synthesized DNA was rapidly degraded. Thus, the methionine analogues did not cause exactly the same affect as the methionine starvation of the met^−^ mutants. There was a difference between the EcoK and EcoB R–M systems and the EcoP1 and EcoP15 R–M systems in their use of SAM, and it was concluded that EcoK and EcoB required SAM for DNA cleavage, whereas EcoP1 and EcoP15 could degrade DNA in the absence of SAM. This further suggested that SAM affected different steps in the reaction mechanism of EcoK and EcoB versus that of EcoP1 and EcoP15.

As cells carrying R–M systems distinct from the EcoK, EcoA or EcoB Type I R–M systems, also destroyed their own DNA on SAM depletion, the EcoP1 and EcoP15 R–M systems were initially grouped with these other systems (known as Type II R–M systems) (11). Hence, although in the earliest phage λ experiments, EcoP1 and EcoP15 behaved like EcoK and EcoB, the dependence on SAM for restriction indicated that they should be classified differently. It was then also realized that the ATP requirements for restriction by EcoP1 and EcoP15 distinguished them from the ATP-independent Type II R–M systems (12,13). Eventually, the designation of EcoP1, EcoP15 and related enzymes as a separate class of R–M systems called Type III R–M systems was proposed by Andrej Piekarowicz in 1978 (10).

### Type III R–M systems from *Haemophilus influenzae*

Just as phage λ revealed R–M in *E. coli*, the HP1c1 phage was used to study R–M systems in *Haemophilus influenzae* (14,15). The HinfIII R–M system was discovered in *H. influenzae* serotype Rf from the extremely low e.o.p. of the HP1c1 phage on this strain (16). Modified phage from *H. influenzae* Re was found to plate efficiently on the Rf strain, suggesting that the host specificities were the same for both. However, the e.o.p. of phage HP1c1 on strain Re was more than that on Rf implying that the former system was less efficient. Other *H. influenzae* strains serotypes, such as Ra, Rb and Rd, showed similar abilities to restrict phage HP1c1 (16,17).

## GENETICS OF TYPE III R–M SYSTEMS

### Methylation-deficient clear plaque mutants of EcoP1 to determine the number of genes in the R–M system

Genetic studies on the control of EcoP1 R–M system showed that, on mutagenesis of *E. coli* P1 lysogens, only r^−^m^−^ or r^−^m^+^ phenotypes could be obtained (18). It was therefore inferred that the r^+^m^−^ phenotype did not exist because it would be lethal to the cell. Scott (19) generated a number of clear plaque mutants of phage P1 in an attempt to elucidate the genes involved in the establishment of lysogeny. These mutant phages of P1 lay in several complementation groups. A cross-streak complementation test was used to classify the mutants into four cistrons, termed c1, c2, c3 and c4. The phenotype of the c2 and c3 complementation groups of clear plaque mutants was elucidated by Rosner (20). These phages were found to have greatly reduced e.o.p. on *E. coli* P1(cry). *E. coli* P1(cry) was isolated as a lysogen that carried a prophage defective in the immunity region but normal with respect to restriction and modification (19). A P1 mutant that was r^−^m^+^ was plated normally on an *E. coli* P1(cry) lysogen, whereas a r^−^m^−^ phage was plated with extremely low efficiency. It was also shown that neither c2 nor c3 mutants could complement r^−^m^−^ lysogens. Hence, it was concluded that the P1 genome itself was subject to modification by the EcoP1 R–M system. The c2 and c3 mutants, being modification deficient, were restricted by the active REase of the *E. coli* P1(cry). Two of the mutations, c2 and c3, formed clear plaques because they failed to modify DNA while retaining the ability to direct active restriction. The phenotype of these clear-plaque c2 mutants of phage P1 was r^+^m. They could not form lysogens because they destroyed the host cell chromosome, but they could go through a lytic cycle because restriction was only expressed late after infection, at times when an infected cell had already lysed. The c2 and c3 mutations were interpreted as being defective in modification and, as they complemented each other, it was thought that two genes were involved in modification. As at least one more gene would be required for restriction, this was taken as support for an ‘at least three gene’ model for P1 restriction and modification. Years later, the two independent c2 alleles were sequenced, and both mutations were found to be in the *mod* gene. Both c2 mutant proteins were purified, and as expected from the *in vivo* phenotype, they had no methylase activity, and their primary defect was that they failed to bind SAM (21). Thus, a ‘two gene’ model was actually sufficient for the Type III R–M systems.

### Identification of res and mod genes of EcoP1 and EcoP15

Normal-sized phage P1 particles contain ∼100 kb of double-stranded DNA (22,23). Mural *et al.* (24) generated fragments of phage P1 using EcoRI and BamHI, and many of these DNA fragments were ligated into appropriate vectors. One such recombinant plasmid containing the 9.2 kb BamHI fragment of P1 DNA expressed both EcoP1-specific restriction and modification. This observation, together with several insertion and deletion mutants of phage P1 (25), enabled Iida *et al.* (26) to define the *res-mod* operon. Iida *et al.* (26) further mapped the BamHI DNA fragment of P1 phage and of a P1-P15 hybrid using various REases. The P1–P15 hybrid phage was a plaque-forming phage derivative in which part of the P1 genome was replaced by DNA sequences from the p15B plasmid (7). Genome mapping showed that several cleavage sites on phage P1 where different from those on the P1–P15 hybrid. Analysis of insertion and deletion mutants of P1 phage and P1–P15 hybrid suggested that the EcoP1 and EcoP15 R–M systems were coded by contiguous genes named *mod* and *res*. Detailed analyses of the BamHI fragment of phage P1 and the P1–P15 hybrid suggested that a 2.2 kb fragment coded for the Mod polypeptide and a 2.8 kb DNA region adjacent to the *mod* gene encoded for the Res polypeptide.

When heteroduplex DNA molecules were prepared from restriction fragments carrying the P1 genes and those carrying the EcoP15 genes, it was found that the two DNA regions were largely homologous. The high degree of similarity between the EcoP1 and EcoP15 systems was expected because it had been shown earlier that mutational defects in one system could be complemented by healthy alleles from the other (27). As one might expect from the heteroduplex analysis of the structural genes for these enzymes, antibodies raised against one of the enzymes could cross-react with both subunits of the other (28). This comparison of the EcoP1 and EcoP15 *mod* genes further showed that the beginning and ends of the *mod* genes were highly homologous, but the central third was totally non-homologous (26,29). As the recognition sequences for these two systems were known to be different, it was suggested that the region of non-homology conferred different sequence specificities (29).

### Identification of Res and Mod subunits of EcoP1 and EcoP15

The subunit functions of EcoP1 and EcoP15 were determined by experiments using an antibody against EcoP15 (30). Western blotting of purified EcoP15 and of a crude extract from a r^−^m^+^ deletion mutant of EcoP15 showed that two subunits were detected in purified EcoP15, but only the smaller one was found in the extract expressing the mutant. The same result was found with EcoP1 and an r^−^m^+^ mutant of P1. Therefore, it was concluded that *res* coded for the larger subunit and *mod* codes for the smaller one.

### Transcription and translation of mod and res

P1 lysogens were shown by Arber *et al.* (31) to manifest methylation activity almost immediately on activation. However, REase activity appeared only 3 h after P1 infection. This suggested that there was a mechanism to protect the host from the action of the REase. The first such effects observed were in a class of streptomycin-resistant strains (*str*), which showed weak EcoP1-specific restriction when in their P1-lysogenic state. The level of residual restriction was dependent on the particular *str* mutation. It was found that *E. coli str* strains carrying a mutation in one of the ribosomal proteins, which reduced spontaneous misreading of codons, were phenotypically r^−^m^+^ (1). Therefore, it was proposed that the synthesis of a functional Res subunit depended on the translational suppression of a stop codon. Two other mutations that affected ribosomal misreading also changed the expression of P1 restriction, supporting the hypothesis that misreading of a stop codon played a role in the expression of the restriction function (32).

Later sequence analysis of the genes showed a 2 bp gap between the end of the *mod* and the beginning of the *res* gene (29). This led to the conclusion that translation of the *res* gene was due to ribosomal shuffling from the terminator to the initiator codon, an event that would be independent of initiation factors. Earlier analysis of the EcoP1 genes led Iida *et al.* (26) to suggest that the EcoP1 genes were expressed from two separate promoters and, in addition, that *res* transcripts could be produced by read-through from the *mod* promoter. However, using northern blotting analysis and promoter studies, Sharrocks and Hornby (33) showed that the EcoP1 *mod* and *res* genes were transcribed separately from their own promoters *in vivo*, and there was no evidence for the production of bicistronic mRNA. Although the *mod* gene was transcribed from two tandem promoters, the *res* gene was transcribed from a promoter present within the *mod* gene. Transcription of *res* may also be regulated through the activity of an antisense promoter within the *res* gene (33).

Redaschi and Bickle (34), in their studies with EcoP1 and EcoP15, showed by western blot analysis that the expression of the Mod subunit positively regulated the amount of Res subunit present in the cell. They postulated that the correct folding of the Res subunit into an active and stable conformation was promoted by its interaction with the Mod subunit, and that the Mod subunit protected the Res subunit from proteolysis by protein–protein interactions. This could explain the sequential expression of the modification and restriction activities of the P1 phage observed by Arber *et al.* (31).

## BIOCHEMICAL CHARACTERIZATION OF TYPE III R–M ENZYMES

### Enzyme purification

Linn and Arber (35) used phage fd DNA to assay for EcoP1 REase activity in their attempt to purify EcoPI. Crude lysates of *E. coli* K(P1) exhibited no apparent activity, but further fractionation gave an enzymatic activity, which specifically cleaved the phage DNA. Meselson and Yuan (12) and Haberman (13) further purified EcoP1 using chromatography and glycerol gradient centrifugation. Eventually Hadi *et al.* (30) expressed and purified both EcoP1 and EcoP15 REases to homogeneity. The purified EcoP1 and EcoP15 proteins showed two bands of 75 kDa and 106 kDa on SDS–PAGE corresponding to the Mod subunit and the Res subunit, respectively (30,36).

Brockes *et al.* (37) described a 500-fold purification of the bacteriophage P1 modification activity from a P1 lysogen of *E. coli*, and the enzyme was later purified to near homogeneity (38). Methylation activity was assayed by measuring the transfer of tritiated methyl groups from SAM to unmodified DNA. This study indicated that the DNA modified *in vivo* by bacteriophage P1 or lambda DNA methylated by P1 MTase were not substrates for cleavage by the EcoP1 REase.

Neither the HinfIII nor the HineI Type III R–M systems have been cloned or sequenced. HinfIII REase was purified from restricting strains using phosphocellulose and DNA-agarose chromatography, and SDS–PAGE analysis revealed two major proteins of 110 kDa and 80 kDa. Gel filtration chromatography analysis of this enzyme preparation indicated that the protein exists as a large complex of the two subunits with a molecular size greater than 200 kDa (10). Another form of HinfIII referred to as HinfIII* was found to copurify. This enzyme had intrinsically bound SAM (39). The SAM free form of the enzyme, referred to as HinfIII could be obtained on storage of HinfIII* after ∼6 weeks.

### Oligomeric status of Type III R–M enzymes

It was originally shown using analytical ultracentrifugation and size exclusion chromatography that Type III REases occur as Res_2_Mod_2_ complexes, whereas the DNA MTases occur as a dimer (Mod_2_) (38,40–42). However, the same techniques recently showed that the EcoP15I REase also exists as an active heterotrimeric Res_1_Mod_2_ complex along with the Res_2_Mod_2_ complex (43). Gupta *et al.* (44) analyzed the EcoP15I Res_2_Mod_2_ and Mod_2_ complexes using small-angle X-ray scattering and analytical ultracentrifugation and showed that although the Mod_2_ complex had a compact shape, the Res_2_Mod_2_ complex adopted an elongated crescent shape. They postulated that Mod_2_ is likely located in the middle of the holoenzyme with a Res subunit at each end of this structure (44).

### The requirement for SAM and ATP for DNA cleavage and modification

The reaction mechanism of the Type III REases was investigated in some detail during late 1970s (45–47). The requirement for SAM for DNA cleavage by EcoP1 (13) and by EcoP15 (47) was studied using partially purified preparations of the REases. SAM was shown to be an allosteric activator with three binding sites on the enzyme. It was shown that the EcoP15 REase formed a complex with unmodified DNA in either the absence or presence of SAM and ATP. Neither the rate nor the yield of complex formation was significantly affected by SAM. Complexes that formed in the presence or absence of SAM could be differentiated on the basis of their stability and their interaction with ATP. Complexes formed with EcoP15 in the presence of SAM were more stable, cleaved DNA at a lower ATP concentration and showed faster kinetics of DNA cleavage following the addition of ATP (46). The role of SAM in the reaction catalyzed by the Type III R–M enzymes is therefore different from its role with the Type I R–M enzymes where it activates the enzyme for DNA binding (48).

DNA cleavage by EcoP1 and EcoP15 REases required ATP and Mg^2+^, but the digestion was incomplete in either the presence or absence of SAM. EcoP1 and EcoP15 REases also methylated unmodified DNA, both in the presence and absence of ATP. In the presence of ATP, the methylation was more efficient, but it acted in competition with the restriction reaction. This was in sharp contrast with the Type I R–M enzymes, which either restrict or modify the DNA but do not do both simultaneously.

It was shown that purified EcoP1 REase and EcoP15 REase exhibited an intrinsic ATPase activity, which was recognition site specific (41,49). Curiously, under the conditions where the EcoKI Type I R–M enzyme hydrolyzed 80% of the input ATP, Reiser and Yuan (45) showed that EcoP15 REase hydrolyzed <1%. When non-hydrolyzable analogs of ATP such as AMP-PNP or ATP-γS were included in the reaction instead of ATP, the EcoP1 REase was unable to restrict pUC18 substrate DNA (41). ATP analogs could substitute for ATP in the cleavage reaction catalyzed by EcoP15 REase, albeit poorly (46). Both forms of the HinfIII enzyme were shown to have an absolute dependence on ATP and a strict requirement for Mg^2+^.

### Recognition sequence determination

Using radiolabeled SAM and λ DNA fragments, it was shown that EcoP1 MTase methylated adenine residues within defined sequences (50). Hattman *et al.* (51) used λ DNA as a substrate for EcoP1 MTase and proposed that the primary product of methylation was the pentameric sequence 5′-AGmACPy-3′ where Py is C or T. In other experiments, SV40 DNA modified by EcoP1 was digested by HaeIII, AluI, HinfI or EcoRII to identify the methylated products (52). Two of the methylated fragments were sequenced. The results were consistent with methylation of the central adenine in the sequence 5′-AGACC-3′. As the EcoP1 recognition sequence is asymmetric and there is no adenine in the complementary strand, it was thought that methylation took place on only one strand. Bachi *et al.* (52) determined that this was the case. The 5′-AGACT-3′ was also modified, albeit poorly. Hadi *et al.* (28) determined the recognition sequence for EcoP15 to be 5′-CAGCAG-3′. Both pBR322 and SV40 DNA were methylated using radiolabeled SAM and EcoP15 REase and the DNA digested with various Type II REases. The separated DNA fragments were subjected to fluorography to find the radiolabeled bands. Meisel *et al.* (53) determined that EcoP15 MTase methylated the second adenine in the recognition sequence. The DNA sequence recognized by HinfIII, and HineI REases was shown to be 5′-CGAAT-3′, making these the first isoschizomers in the Type III R–M systems (54). Modification of DNA by HinfIII was found to occur at the second adenine of the recognition sequence and was confined to one strand (55). Ten Type III R–M enzymes have now been characterized biochemically (Table 1). All recognition sequences are five or six bases in length and are asymmetric.

### Identification of the cleavage site on DNA

Bacteriophage λ DNA was first shown to be broken by EcoP1 REase into fragments of high molecular weight (12). Adler and Nathans (56) observed that cleavage of SV40 DNA by EcoP1 REase converted the DNA to linear molecules of similar length. Risser *et al.* (57) reported that EcoP1 REase prepared from a P1 lysogen of *E. coli* made one double-strand break in SV40 DNA. In the presence of SAM and ATP, the enzyme cleaved 70% of the closed circular SV40 DNA molecules to produce linear molecules and rendered the remaining 30% resistant to further cleavage. It was also shown that linear SV40 DNA molecules cleaved by EcoP1 REase were not cleaved at a unique site. The EcoP1 REase cleavage sites on SV40 DNA were mapped relative to the partial denaturation map and to the EcoRI and HpaII REase cleavage sites. These maps suggested there were a minimum of four unique but widely spread cleavage sites. Furthermore, this study clearly showed that the locations of the EcoP1 REase cleavage sites and methylation sites overlapped. To map the site of cleavage, Bachi *et al.* (52) cleaved SV40 DNA by EcoP1 followed by EcoRI, HpaII, BamHI and confirmed that SV40 DNA has at least five potential cleavage sites. These studies showed that the cleavage sites were unique, and EcoP1 cleaved DNA 25–27 base pairs away from the recognition sequence.

Hadi *et al.* (28) isolated a 434 bp HaeIII fragment from pBR322 DNA, which contained the EcoP15 recognition site 70 bp from the end of the fragment. The fragment was radiolabeled, digested with EcoP15 and analyzed on a 6% polyacrylamide gel. In addition to the large amount of radioactivity present at the position of the original fragment, a band of ∼40 bp long was seen. It was argued that as the EcoP15 recognition sequence in this fragment was situated 70 bp from one end, the DNA was cut ∼30 bp 3′ to the CAGCAG sequence. Furthermore, in the same study, it was shown that EcoP15 produced a 5′ single-stranded protrusion on cleavage of the DNA.

EcoP1, EcoP15 and HinfIII REases cleave the DNA ∼25–27 base pairs downstream of the recognition sequence to leave ends that have 2–3 nt single-stranded 5′-extensions (28,52,58). It was suggested that the distance of ∼9 nm between recognition site and cleavage site could easily be spanned by the enzyme without invoking movement along the DNA. Early experiments showed a tendency for the enzyme to cleave adjacent to thymidine residues in the DNA. However, this was not substantiated by later work (28,46,52), which suggested that a likely model for the interaction was that the Mod subunit recognized and bound to the specific sequence and directed the binding of the Res subunit to the site that would be cleaved. According to this model, the Res subunit need not necessarily have a high affinity for DNA.

### Identification of requirement for two recognition sites for successful DNA cleavage

In addition to discovering the two forms of HinfIII REase, Piekarowicz and coworkers were the first to report what has now been accepted as the distinguishing features of Type III restriction enzymes (10). These workers showed the requirement of more than one site for DNA cleavage by these enzymes. Definitive evidence for the requirement of more than one site came from an experiment where supercoiled and linear DNA substrates were used to study both restriction and modification. Although methylation was not affected, the restriction of supercoiled DNA gave rise to linear fragments both in the absence and presence of SAM (58). The HinfIII-linearized ColE1 DNA was further denatured, renatured and analyzed on agarose gel to determine the site of cleavage by HinfIII REase. Agarose gel electrophoresis of the renatured ColE1 DNA showed that this DNA migrated predominantly at the position of the nicked circular form of ColE1 DNA, indicating that the cleavage of ColE1 DNA was not at a fixed site. This conclusion was further confirmed using ColE1 DNA linearized by HinfIII and subsequently cleaved with EcoRI or SmaI. ColE1 DNA contains a single site for these enzymes, but their use on HinfIII-linearized ColE1 DNA resulted in 10 fragments, indicating the presence of at least five potential cleavage sites for HinfIII REase. As DNA was cleaved only once by HinfIII, both in the presence and absence of SAM, and cleavage was not observed with DNA containing a single site, these authors suggested that HinfIII would require the presence of multiple recognition sites for efficient DNA cleavage (58). Later, Piekarowicz (39) demonstrated that HinfIII cleavage depended on the number of recognition sites present on the DNA substrate.

### Recognition site orientation requirement for successful DNA cleavage and the strand bias model

All Type III REases recognize asymmetrical DNA sequences and the modified DNA bears methyl groups in only one strand of the DNA. It was recognized that the asymmetrical methylation of modified DNA posed a conceptual problem. When a modified recognition site is replicated, the single methyl group is inherited by one daughter DNA molecule, which remains completely modified, whereas the same site in the other daughter molecule is completely unmodified and ought to be subject to restriction, which would be lethal for the cell (27).

To avoid this problem, Type III R–M enzymes cleave DNA with two sites arranged in opposite orientation. This became clear when it was observed that Phage T3 DNA was restricted by EcoP15, whereas the DNA of its close relative T7 was not. Although phage T7 DNA has 36 sites for EcoP15, all have the same orientation along the DNA, whereas the T3 DNA contains both orientations of the EcoP15 site. These observations led to the hypothesis that restriction requires two recognition sites that have to be in inverse orientation and sites in direct orientation would not be subject to restriction but could still be modified (59). The strand-bias model therefore offers an explanation of how suicidal restriction by EcoP15 or EcoP1 REases is prevented. This prediction was later tested by using phage M13 DNA constructs with arrangements of EcoP15 sites in both direct and inverse orientations. Only sites in inverse orientation were restricted, although any combination of sites (or a single site) could be modified (60). Thus, Type III REase recognition sites are ‘symmetrical’ and interrupted at the center of symmetry by a non-specific spacer of variable length. This model explained several early observations concerning DNA cleavage by Type III REases. For instance, after growing density-labeled EcoP1-modified phage DNA for one cycle on a non-P1 modifying host, phages of intermediate density containing DNA with one P1-modified parental DNA strand and one unmodified newly synthesized DNA strand were isolated. The DNA of these phages was totally resistant to EcoP1 restriction (1).

### Cleavage of unusual DNA substrates and exceptions to the strand-bias model

The requirement for two inversely oriented unmodified sites for cleavage by Type III REases was consistent with later studies (40,61). However, it has been observed that other site orientations can be cleaved. It is generally thought that the enzyme concentration, the nature of monovalent cations in the reaction buffer and the flanking sequences of the recognition sites seem to affect the cleavage patterns by Type III REases (62).

Type III restriction enzymes have been demonstrated to cleave DNA with a single recognition site on a linear DNA, albeit less efficiently compared with DNA with two sites in head-to-head orientation (63,64). Blocking the ends of DNA substrates by streptavidin on a linear DNA having sites in head-to-head orientation increased cleavage events, suggesting the enzyme molecules loaded on the DNA might need a free end. Linear DNA with sites in a tail-to-tail orientation where also cleaved if the ends were capped by streptavidin (64). The blocking of DNA ends on a linear substrate with sites in head-to-head or tail-to-tail orientation was suggested to prevent the release of the enzyme from the DNA and consequently enhance translocation of the enzyme back and forth until it interacted with another enzyme molecule (64). Mucke *et al.* (61) studied the effect of distance between two inversely oriented recognition sites and showed that Type III REases also efficiently cleave two adjacent head-to-head or tail-to-tail sites. It was also shown that substrates with sites in the same orientation on a DNA molecule (49,60) or single site on close circular (40) or catenated substrates with one site per catenane ring (65) were not cleaved.

Type III R–M enzymes remain bound to the DNA after they cut DNA 25–27 bp away from the recognition site. Raghavendra and Rao (66) showed with EcoP15 REase that the intact recognition site on the cleaved DNA sequestered the REase, thus decreasing the effective concentration of the enzyme. However, when the restriction assay was performed in the presence of exonucleases, the removal of cleaved DNA by exonucleases released EcoP15 REase to perform further rounds of catalysis.

### Cleavage models invoking DNA translocation (tracking) and looping

In 1992, work from the laboratories of Tom Bickle and Detlev Kruger clearly demonstrated that *in vitro* restriction of a covalently closed circular DNA, which has three EcoP15 sites in the same orientation, showed no DNA cleavage. However, when any one of these sites was altered so that at least two adjacent sites were in a head-to-head orientation, the DNA was linearized (60). This cleavage did not depend on the distance between the sites and this led to the collision-cleavage model for the Type III REases. Later, in an elegant experiment, DNA cleavage was studied in a DNA substrate where a Lac repressor-binding site was flanked by two properly oriented recognition sequences. DNA was found cleaved only in the presence of IPTG, which caused the Lac repressor to dissociate from the DNA. This observation suggested that DNA is looped through the enzyme bound to a cognate sequence, ATP hydrolysis providing the energy for such a ‘tracking’ mechanism (49).

The basis of long-distance communication between sites, for which a low level of ATP hydrolysis was required, was not accurately defined in the collision-cleavage model and more models have been recently proposed (67,68). The first model invoked DNA looping and limited translocation (69–71), whereas the second invoked ATP-triggered passive diffusion on the DNA (64,65,72,73). Reich *et al.* (69) visualized the DNA loop structures on linear DNA molecule with EcoP15 in the presence of ATP and metal ions using atomic force microscopy. The DNA looping model was further elucidated in later AFM studies (70,71), but it has been questioned using the results of single molecule imaging studies, which showed that Type III REases can communicate between their sites in a 1D manner without loop formation (64,65,72). An alternative mechanism in which DNA sliding is activated by ATP hydrolysis was proposed in which the REase remains bound to DNA via weak electrostatic interactions and slides along it until it encounters another REase bound at a distant site (72,73). These two different single molecule techniques lead to conclusions that are in disagreement. DNA looping and movement of the enzyme along a DNA contour are both fast events occurring on the millisecond timescale (67), which push the single molecule methods to their limits. In addition, the variable Res_1_Mod_2_ (43) or Res_2_Mod_2_ (44) stiochiometries may also be a source of the apparent disagreements as the compositions are rarely checked. At some point in the future, the disagreement between the two techniques may be reconciled.

## NEW OBSERVATIONS ON THE GENETICS OF TYPE III MTASES AND NEW TECHNOLOGICAL APPLICATIONS OF TYPE III REASES

### Phase-variable Type III MTases

Several host adapted bacterial pathogens have the *mod* genes associated with Type III R–M systems. The *mod* gene contains tandem repeats that are prone to phase variation (*Pasteurella haemolytica*, *Haemophilus influenzae*, *Helicobacter pylori*, *Neisseria gonorrhoeae* and *Neisseria meningitidis*) (74–78). Phase variation is an adaptive process by which pathogenic bacteria undergo frequent and reversible phenotypic changes in expression of surface antigens such as lipopolysaccharide and outer-membrane proteins resulting from genetic alterations in specific *loci* of their genomes. This helps in colonization of the host, adaptation to host environments and evasion of immune responses. The number of repeats within the *mod* gene influences the rate of phase variation and expression of the *mod* gene. Owing to mutations within the *res* gene, the REase may not be functional. Therefore, the evolution of Type III R–M systems into epigenetic modulators for controlling gene expression results in the loss of the DNA restriction function. The phase variable Type III R–M systems may epigenetically regulate genes through differential methylation of the genome. Furthermore, a phase-variable MTase could be involved in pathogenicity by randomizing virulence factor expression through global changes in methylation (78).

### Applications of Type III REases

The distance of 24–28 bp between the recognition and cleavage sites in the DNA molecule by a Type III REase is the longest defined distance found so far for any REase. Therefore, the EcoP15 REase has been used as the tagging enzyme in Serial Analysis of Gene Expression (SAGE) of transcriptome experiments. SAGE originally used a Type IIS restriction enzyme as the tagging enzyme, but these only generate tags of 15 bp and were only useful in analyzing genes from lower organisms that contain fewer genes. As EcoP15I generates 26 bp tags on digestion, it has been successfully used as the tagging enzyme in SuperSAGE assays. As SuperSAGE is based on the extraction of DNA fragments of defined length, it would be a great benefit to direct cleavage by EcoP15 REase to exactly that recognition site located in the sequence tags. These results could be potentially exploited in the high-throughput quantitative transcriptome analysis (79). Along with generating long fragments, the other advantage is that EcoP15 cleavage generates a 2 nt overhang at the 3′ terminus, which is easy to fill in. The increased tag length of SuperSAGE (26 bp) dramatically improves the efficiency of identification of genes corresponding to the tags. In model organisms, for which genomic or cDNA databases are available, 26 bp tags allow almost perfect gene annotation by BLAST searches against sequence databases. This was not possible in the original SAGE, whereby ambiguous tag-to-gene annotation was an inherent problem. The cleavage activity of EcoP15 in presence of sinefungin has provided a powerful tool for the generation of 26 bp long cDNA tags during SAGE assays (80), and using this principle, a commercial kit called SOLid SAGE has been developed by Applied Biosystems, USA.

Lastly, the cleavage of fluorescence-labeled DNA substrates by the EcoP15 REase has been successfully used in determining the number of CAG triplet repeats present in the Huntington’s disease gene (30–180 CAG repeats on chromosomes of patients as compared with 6–37 repeats on chromosomes of unaffected individuals) (81).

## CONCLUSIONS

Despite all of the work described earlier in the text, many aspects of the biology and reaction mechanism of the Type III R–M systems remained mysterious. For example, why are these nucleases relatively inefficient *in vitro*? One would predict from the properties of the isolated enzymes that restriction *in vivo* by Type III REases would be relatively inefficient. However, this is not at all the case. Restriction by the EcoP1 R–M system of phage λ, for example, is more efficient than any of the other Type I and Type II R–M systems that can be found in *E. coli* (1). As replication generates completely unmodified sites, how does the cell avoid restriction at these sites?

Type III R–M systems have been used as model systems to study various aspects of protein–DNA interactions. Although there has been a lot of progress in this area in the past decade, several questions yet remain to be answered especially regarding the mechanism of catalysis and site-to-site communication. It is also of interest to know why these enzymes are more complex than the Type II R–M enzymes. Perhaps the complex R–M systems like the Type I and III should be thought of as enzymes relevant to DNA replication, repair and recombination. Further advances in our understanding of these enzymes will surely come from structural analysis, genome sequence analysis and single molecule studies.
